# Rocking block simulation based on numerical dissipation

**DOI:** 10.1007/s11071-024-09974-1

**Published:** 2024-07-26

**Authors:** A. M. D’Altri, G. Vlachakis, S. de Miranda, P. B. Lourenço

**Affiliations:** 1 Department of Civil, Chemical, Environmental, and Materials Engineering, University of Bologna, Bologna, Italy; 2 Department of Civil Engineering, ISISE, ARISE, University of Minho, Guimarães, Portugal

**Keywords:** Rocking, Dynamics, Masonry, Out-of-plane collapse, Finite element method, Cultural heritage structures

## Abstract

In this paper, a computational approach based on numerical dissipation is proposed to simulate rocking blocks. A rocking block is idealized as a solid body interacting with its foundation through a contact-based formulation. An implicit time integration scheme with numerical dissipation, set to optimally treat dissipation in contact problems, is employed. The numerical dissipation is ruled by the time step and the rocking dissipative phenomenon at impacts is accurately predicted without any damping model. A broad numerical campaign is conducted to define a regression law in analytic form for the setting of the time step, depending on the block size and aspect ratio, the contact stiffness, as well as the coefficient of restitution selected. The so-obtained regression law appears accurate and an a posteriori validation with cases not in the training dataset confirms the effectiveness of the approach. Finally, the comparison with available experimental tests highlights the approach efficacy for free rocking and harmonic loading cases (in a deterministic sense), and for earthquake-like loading cases (in a statistical sense). It is found that rocking blocks with sizes of interest for structural engineering (e.g., cultural heritage structures) can be simulated with time steps within 10^–3^ ÷ 10^–1^ s, so allowing very fast computations.

## Introduction

In the last decades, rocking structures have been intensely investigated, and various models to predict the rocking motion have been developed. On the one hand, this was motivated by the need of analyzing the dynamic response of existing and cultural heritage structures, e.g., masonry and dry-stone walls [1–5], stone monuments [6–9], as well as statues [10, 11], that typically experience damage/collapse due to seismic events. On the other hand, rocking structures attracted the attention of researchers as they might be used as seismic design strategies [12], given that the uplift of a rocking block limits the design forces acting in the superstructure, as well as in the foundation. This “rocking isolation” strategy can be used on both buildings [13] and bridges [14].

One significant issue with the seismic response of a rocking block is the large sensitivity to its defining features, i.e. minor changes in rocking block properties may result in significantly different time-history responses. Indeed, experiments involving dynamically excited rocking specimens are rarely replicable, and the response is typically labeled as chaotic [12] (i.e., nonreproducible and unpredictable). Accordingly, a plausible approach to proceed with model validation, instead of the classical approach of comparing deterministically the specimen and the model responses under a specific ground excitation, should be based on statistical validation (as proposed in [15]).

The most established model to predict the response of a rocking block has been introduced by Housner [16]. Such well-known analytical model, even though the solution is typically obtained numerically given the event-by-event formulation, represents the so-called classical rocking theory, based on the hypotheses of (i) rigid block and rigid foundation, (ii) two potential contact points, (iii) no sliding, (iv) no bouncing, and (v) energy dissipation at impacts. Based on the classical rocking theory [16], several enhancements and extensions [17–37] have been developed to treat a wide range of rocking structures with a multitude of different boundary conditions. However, the hypothesis of no sliding (as well as no bouncing) might be too strict for many actual applications, as sliding (and bouncing) is not always prevented. For this reason, more general analytical models accounting also for sliding (as well as bouncing) have been proposed [38–48]. Nonetheless, most of these models did not find widespread actual applications given the complexities and limitations in the generalization of the problem.

In this context, numerical approaches may represent an appealing choice to generalize the solution for rocking problems, as they are able to deal with complex geometries, boundary conditions, and mechanical aspects (such as sliding, bouncing, 3D effects, material nonlinearities etc.). When considering masonry and cultural heritage structures, the use of block-based numerical models [49] also allows to account for the actual masonry pattern as well as the interaction with adjacent structural elements. In this framework, the adoption of contact-based numerical approaches appears particularly appropriate to model rocking blocks. The explicit time integration scheme has been typically preferred, see e.g. applications within the so-called discrete element method (DEM) [50–56]. The main drawback of these contact-based explicit approaches consists in the definition of a suitable damping model. Indeed, the choice and the characterization of a damping model (e.g., Rayleigh damping) is challenging and non-univocal [50], as the rocking block response is nonlinear, and a representative frequency of the rocking motion cannot be defined univocally. Accordingly, the setting of the damping model is mostly conducted to fit some reference response in a phenomenological fashion, rather than having a clear physical meaning. To bridge the gap between classical rocking theory and numerical models, in terms of energy dissipation, an equivalent viscous damping model calibrated on analytical solutions has been proposed in [57].

The adoption of implicit time integration schemes to model rocking blocks has found less interest in the scientific community, as such schemes are typically characterized by numerical (i.e., algorithmic) dissipation [58, 59], and so the response depends on the time step chosen [60, 61]. Indeed, very small time steps should be adopted to reduce the amount of numerical dissipation that would lead to inconvenient simulations. However, by noticing that when dealing with rocking motion the setting of damping models appears as questionable as relying on numerical dissipation only, this paper investigates the possibility of utilizing an implicit time integration scheme with numerical dissipation without any damping model to simulate rocking blocks. In other words, the use of numerical dissipation to account for the rocking energy dissipation does not appear more problematic than ad hoc calibrated damping models, and it leads to superior computational efficiency. The potential benefit of this choice, beyond its simplicity, is indeed immediately clear: if only numerical dissipation is employed, rather large time steps can be used, so allowing very fast and convenient computations.

In this framework, a pioneering approach was proposed in [62] where rocking blocks were modelled by means of beam elements with no-tension zero-length fiber cross-sections representing the rocking surfaces, using a corotational formulation to account for geometric nonlinearity. In particular, the adoption in [62] of the well-known implicit time integration scheme with numerical dissipation proposed in [58], also known as HHT or α-method, and quasi-rigid rocking surfaces allowed to obtain classical rocking solutions through numerical dissipation only, without having a strong dependency on the adopted time step (which could vary between 0.0001 s and 0.001 s), yet without directly controlling the energy dissipation.

In this paper, a rocking block is idealized as a solid body interacting with its foundation through a contact-based formulation. The HHT time integration scheme is employed with an algorithmic setting to optimally treat dissipation in contact problems [63]. The rocking dissipative phenomenon at impacts is investigated, correlating its dependency on the time step. A broad numerical campaign is conducted to define a regression law in analytic form for the setting of the time step, using as reference the classical rocking theory. Comparisons with available experimental tests are used to check the efficacy of the regression law.

The paper is structured as follows. Section 2 discusses the modelling assumptions at the basis of the present computational approach. Section 3 presents the strategy adopted to define a suitable setting of the time step, as well as the obtained regression law together with its post validation. Section 4 shows the comparison with available experimental tests, particularly free rocking and harmonic loading cases (in a deterministic sense) from [64], and earthquake-like loading cases (in a statistical sense) from [15].

## Modeling of rocking blocks

In this section, the classical approach to model a rigid rocking block according to [16], used as reference, is firstly briefly recalled. Then, the proposed computational approach based on numerical dissipation to model a solid deformable body interacting with its foundation though a contact-based formulation is discussed together with few details about the adopted time integration method.

### Classical rocking theory

**Fig. 1:**
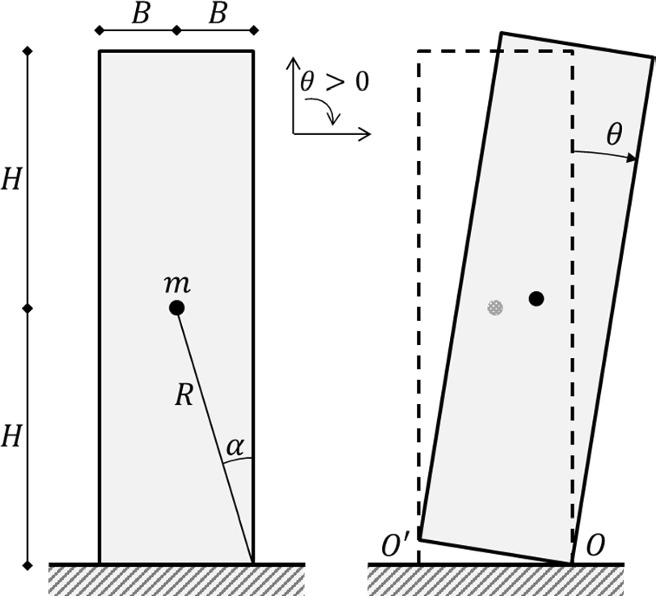
The rocking block

According to the classical rocking theory [16], the symmetric rocking block (Fig. 1) is idealized as a rigid body, characterized by the semi-diagonal $R$ and slenderness $\alpha = {\text{atan}}\left( {B/H} \right)$, rocking on a rigid foundation. The hypotheses of no sliding and no bouncing yield.

The equation of motion of the rigid block in in-plane free rocking about pivot points $O$ and $O^{\prime}$, and measured using the rocking angle $\theta$ (Fig. 1) is:

1 $$\ddot{\theta }\left(t\right)= -{p}^{2}\left\{ \text{sin}\left[\alpha \text{sgn}\left(\theta \left(t\right)\right)- \theta \left(t\right)\right]\right\}$$

where $p=\sqrt{(mgR/{I}_{0})}$ is the frequency parameter of the rigid rocking block, with $m$ representing the mass of the block, and ${I}_{0}$ representing the rotational moment of inertia with respect to the pivot points. For rectangular cuboid blocks, ${I}_{0}=\left(4/3\right)m{R}^{2}$ and, hence, $p=\sqrt{(3g/4R)}$.

Impacts between the block and the foundation occur when $\theta =0$. At any impact, the pivot point changes and the rotation changes sign. Importantly, impacts result in instantaneous energy losses. According to [16], the reduction of energy at any impact may be described using the coefficient of restitution $r$, defined as the ratio of postimpact to preimpact kinetic energy. Furthermore, within the preceding assumptions, the classical rocking theory [16] provided an estimation of the coefficient of restitution by employing the conservation of angular momentum:

2 $$r={\left[1-\frac{m{R}^{2}}{{I}_{0}}\left(1-\text{cos}2\alpha \right)\right]}^{2}$$

It should be underlined that, although various more recent formulations (see e.g. [31, 57, 65]) adopt as coefficient of restitution $\sqrt{r}$, i.e. the ratio of the pre- and post-impact angular velocities, the original definition of ratio of kinetic energies is herein considered.

In free rocking motion, maximum rocking angles ${\theta }_{\text{n}}$ and half rocking periods ${T}_{\text{n}}/2$ along with the number of impacts $\text{n}$ are defined, according to [16], as:

3 $$\begin{aligned} & {\theta }_{\text{n}}=1-\sqrt{1-{r}^{\text{n}}\left[1-{\left(1-{\theta }_{0}\right)}^{2}\right]} , \\ &  {T}_{\text{n}}/2=\frac{2}{p}{\text{tanh}}^{-1}\sqrt{{r}^{\text{n}}\left[1-{\left(1-{\theta }_{0}\right)}^{2}\right]} \end{aligned}$$

where ${\theta }_{\text{n}=0}={\theta }_{0}$ is the initial rocking angle, and ${T}_{\text{n}=0}/2={T}_{0}/2$ is computed by doubling the time elapsed until the first impact. In the following, Eqs. (1) and (3) are referred to as analytical solutions. As a result, the rocking behavior is nonlinear due to (i) the change of pivot point (from $O$ to $O^{\prime}$ and vice versa) and (ii) the jump discontinuity of the angular velocity pre- and post-impact caused by the impact energy dissipation, ruled by the coefficient of restitution. Further insights on the nonlinear nature of the rocking behavior can be found in [19, 66–70].

### Numerical modeling

**Fig. 2:**
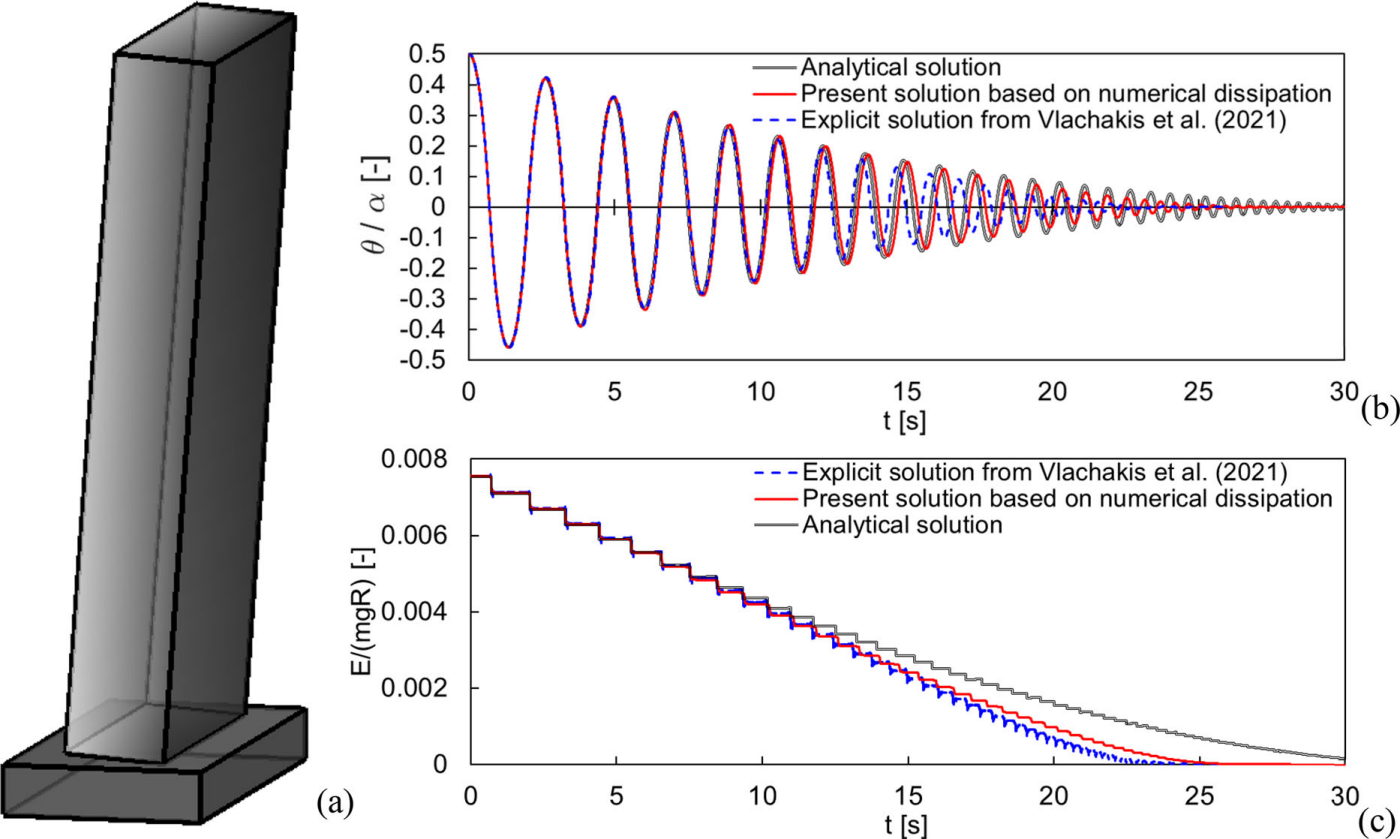
Proof of concept for free rocking response. **a** Solid block rocking on its foundation. Comparison of free rocking response in terms of **b** normalized rocking angle time history, and **c** normalized total energy time history (being ${\text{E}}$ the total energy), for a block with $2H=4.2 m$, $2B=0.6 m$, $r=0.94$, ${\theta }_{0}/\alpha =0.5$ (the present solution has been obtained with ${k}_{n}=5$e + 08 N/m^3^, $\Delta t=0.013$ s)

A free-standing rocking block is idealized as a solid deformable body interacting with its foundation (Fig. 2a) though a contact-based formulation, characterized by a finite contact stiffness ${k}_{n}$. The Young’s modulus of the block is assumed so that the overall block stiffness is much higher than the contact stiffness, i.e., the block Young’s modulus becomes irrelevant to the present study. Additionally, a reasonably high value of friction coefficient prevents sliding to occur. In other words, the contact stiffness ${k}_{n}$ is the only mechanical parameter which has a direct and significant effect on the rocking behavior of the block.

The one-step implicit time integration method with numerical dissipation developed in [58], also called HHT method (as well as $\alpha$-method), is considered. The HHT method approximates the solution of an undamped structural dynamics problem by means of the following relationships:

4 $$\begin{aligned} & {\mathbf{M}}\ddot{\varvec{u}}_{{{\text{i}} + 1}}  + \left( {1 + \alpha _{{HHT}} } \right){\mathbf{K}}\varvec{u}_{{{\text{i}} + 1}}  - \alpha _{{HHT}} {\mathbf{K}}\varvec{u}_{{\text{i}}}  = {\mathbf{F}}_{{{\text{i}} + 1}}  \\& {\text{ }}\varvec{u}_{{{\text{i}} + 1}}  = {\text{ }}\varvec{u}_{{\text{i}}}  + \Delta t\dot{\varvec{u}}_{{\text{i}}}  + \Delta t^{2} \left[ {\left( {1/2 - \beta _{{HHT}} } \right)\ddot{\varvec{u}}_{{\text{i}}}  + \beta _{{HHT}}\ddot{\varvec{u}}_{{{\text{i}} + 1}} } \right] \\ & {\text{  }}\dot{\varvec{u}}_{{{\text{i}} + 1}}  = {\text{ }}\dot{\varvec{u}}_{{\text{i}}}  + \Delta t\left[ {\left( {1 - \gamma _{{HHT}} } \right)\ddot{\varvec{u}}_{{\text{i}}}  + \gamma\ddot{\varvec{u}}_{{{\text{i}} + 1}} } \right] \end{aligned} $$

For $\text{i}=\text{0,1},2,\dots$, being $\Delta t$ the time step, and${\alpha }_{HHT}$, ${\beta }_{HHT}$, and ${\gamma }_{HHT}$ parameters governing the numerical dissipation and the stability of the algorithm (the subscript HHT has been added to avoid any confusion with other parameters). In Eq. (4), $\mathbf{M}$ is the mass matrix, $\mathbf{K}$ is the stiffness matrix, $\mathbf{F}$ is the external forces vector, and ${\varvec{u}}$ is the displacement vector (superimposed dots symbolize time differentiation). The solution is initiated through initial conditions ${{\varvec{u}}}_{0}={\varvec{u}}(0), \, {\dot{{\varvec{u}}}}_{0}=\dot{{\varvec{u}}}(0), \, {\text {and}} \, {\ddot{{\varvec{u}}}}_{0}={\mathbf{M}}^{-1}\left(\mathbf{F}\left(0\right)-\mathbf{K}{{\varvec{u}}}_{0}\right)$.

To optimally treat numerical dissipation in the elastodynamic contact problem, the setting of ${\alpha }_{HHT}$, ${\beta }_{HHT}$, and ${\gamma }_{HHT}$ is carried out according to [63]. In particular, the time integration parameters are adopted to ensure unconditional stability, second-order accuracy, momentum transfer in dynamic rigid impact problems and optimal numerical dissipation, [62] as:

5 $${{\alpha }_{HHT}=-\sqrt{2}+1=-0.41421, {\beta }_{HHT}=1/2=0.5, \gamma }_{HHT}=\sqrt{2}-1/2=0.91421$$

Accordingly, once defined ${\alpha }_{HHT}$, ${\beta }_{HHT}$, and ${\gamma }_{HHT}$, numerical dissipation is fully governed by the time step $\Delta t$. The higher the $\Delta t$, the higher will be the numerical dissipation.

### Proof of concept

The possibility to find a certain time step $\Delta t$ that guarantees good estimates of the rocking response is shown in Fig. 2. The free rocking response of a solid block (Fig. 2a) with initial rocking angle ${\theta }_{0}/\alpha =0.5$ obtained by the present solution is compared, in terms of normalized rocking angle (Fig. 2b) and normalized total energy (Fig. 2c), with the analytical solution given by Eq. (1) and the explicit numerical solution from [57]. It can be observed that the free rocking response is accurately reproduced by the present solution and the step-like rocking dissipative phenomenon at impacts is well predicted, even without any damping model.

It should be pointed out that the solution in Fig. 2 has been obtained with a rather large time step (in this case $\Delta t=0.013$ s), which thus allowed a very fast simulation (416 s on a commercial laptop) given the limited number of increments needed.

**Fig. 3:**
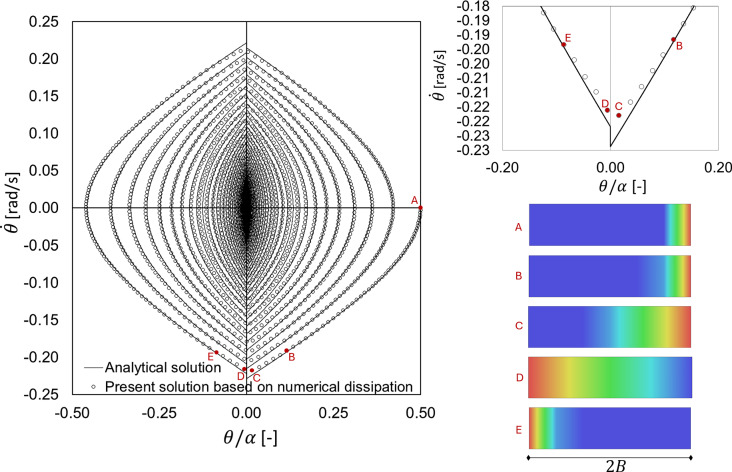
Phase portrait for the case shown in Fig. 2. Comparison between the analytical and the present solutions (left). Magnified phase portrait at the first impact (top right). Contact pressure contour plots, from red (maximum contact pressure) to blue (no contact), at subsequent instants

Further features of rocking response of the present solution shown in Fig. 2 can be gathered by observing the phase portraits in Fig. 3. Indeed, the phase portraits show an overall good agreement between the analytical and the present solutions (see Fig. 3, left). In particular, it appears worth to highlight the response around impacts (see, e.g., the first impact in Fig. 3, top right). On the one hand, the analytical solution shows a sharp jump of the angular velocity at impact, fully governed by the coefficient of restitution. On the other hand, the present solution shows a smoother response at impacts, given the presence of a deformable contact interface. Indeed, the contact pressure distribution (see Fig. 3, bottom right) at initial condition (A) starts to sensibly change at the instant (B), i.e., the instant in which analytical and numerical solutions tend to drift apart. The first impact happens between (C) and (D), where in both cases the contact pressure is rather distributed on a considerable portion of the contact interface, with the maximum contact pressure observed in opposite corners. From the instant (E) the analytical and numerical solutions tend to overlap again. Although globally in agreement, the numerical solution represents the impact in a smoother way than the analytical one, the latter based on the hypothesis of only two potential contact points.

**Fig. 4:**
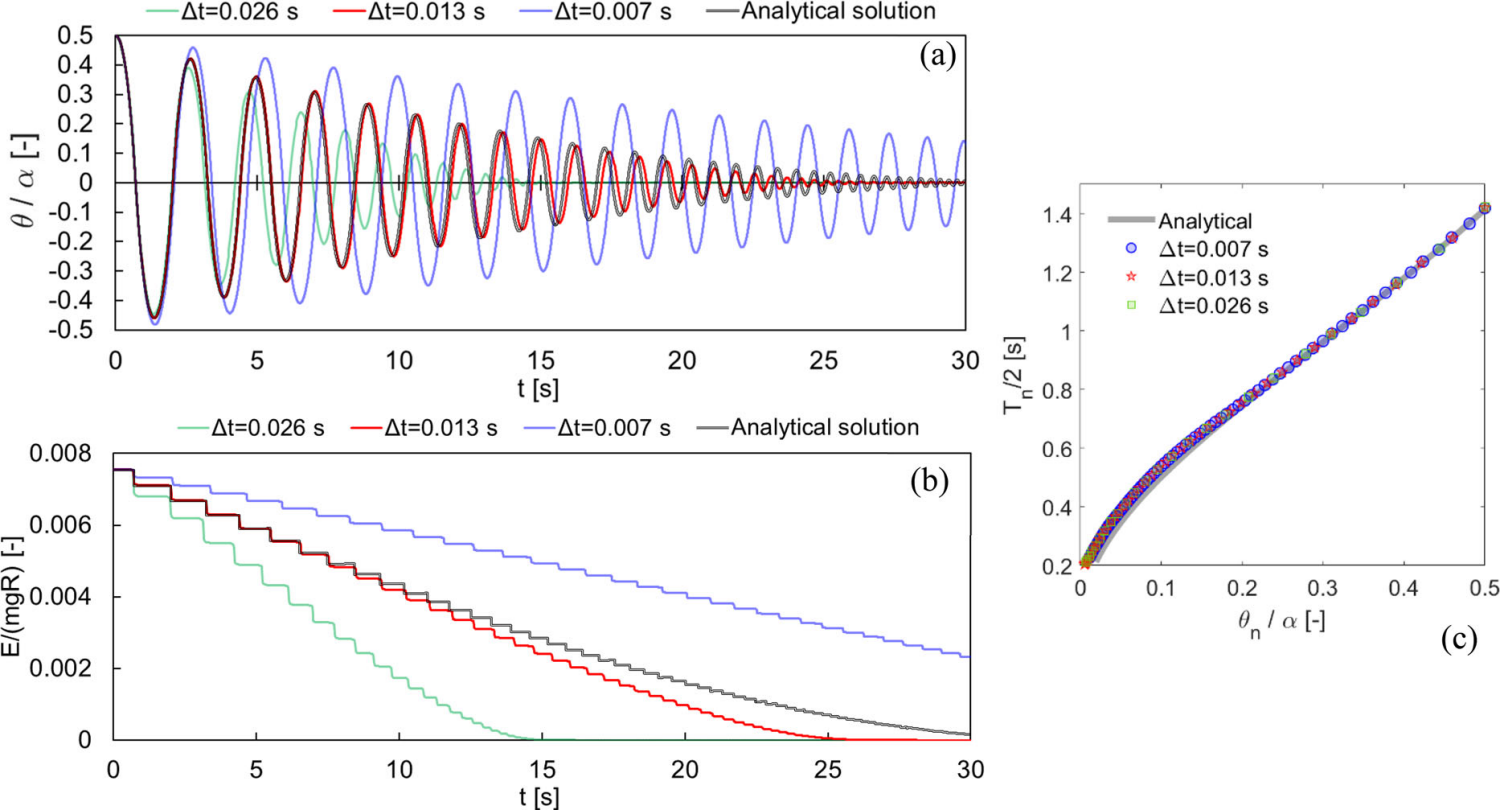
Effect of the time step on the present solution based on numerical dissipation (see Fig. 2 for the settings). Comparison of the present solution ($\Delta t=0.013$ s) with two other time steps ($\Delta t=0.026$ s and $\Delta t=0.007$ s) in terms of **a** normalized rocking angle time history, **b** normalized total energy time history, and **c** half rocking period versus normalized rocking angle diagram for subsequent impacts

The effect of the time step on the present solution is shown in Fig. 4 where the previously selected time step $\Delta t=0.013$ s and the analytical solution are compared with different time steps, i.e. $\Delta t=0.026$ s and $\Delta t=0.007$ s. As it can be noted, the time step has a direct effect on the energy dissipation, leading to normalized rocking angle time histories sensibly different (Fig. 4a). This effect is clearly shown in Fig. 4b, where the normalized total energy time histories show larger energy drops at impacts for larger time steps. Interestingly, the half rocking period versus normalized rocking angle diagram for subsequent impacts shown in Fig. 4c highlights that the present modelling strategy is able to accurately represent the nonlinear period-to-amplitude relationship as in Eq. (3), independently from the time step utilized. Indeed, the time step only rules the energy losses at impacts and therefore the distance between the points in the period-to-amplitude diagram. Consequently, the three cases considered in Fig. 4c lay on the same curve. As in the classical rocking theory the distance between the points in the period-to-amplitude diagram is governed by the coefficient of restitution in Eq. (2), it appears that the time step could be tuned to guarantee the desired energy dissipation at impacts.

**Fig. 5:**
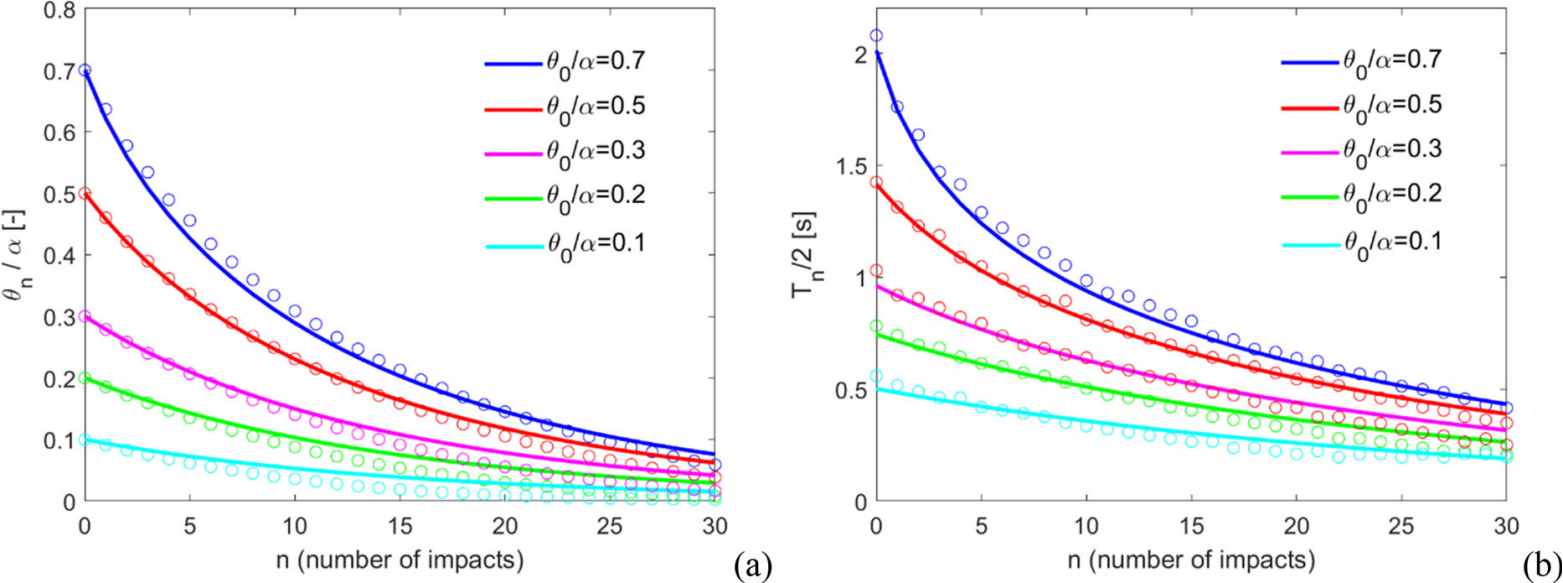
Free rocking response for different values of initial amplitude (${\theta }_{0}/\alpha$) with the same $\Delta t$, in terms of **a** normalized rocking angles and **b** half rocking periods, along with the number of impacts. Solid lines represent the analytical solution, while hollow circles represent numerical solutions

The free rocking response of the same block for different values of initial amplitude (${\theta }_{0}/\alpha$) with the same $\Delta t$ is compared with the analytical solution given by Eq. (3) in Fig. 5, in terms of normalized rocking angles (Fig. 5a) and half rocking periods (Fig. 5b), along with the number of impacts. As it can be observed, the problem appears amplitude independent, i.e. the same $\Delta t$ can be utilized independently of the rocking angle. This feature appears particularly appealing, and guarantees a reasonable generalization of the present computational approach.

As a result, the surgical use of the numerical dissipation of the time integration scheme allows the modelling of energy dissipation at impacts in a phenomenological manner, allowing fast numerical simulations. In other words, the proposed modelling strategy permits to account for the desired energy dissipation while using the largest possible time step.

## Setting of the time step

In this section, a strategy for the setting of the time step $\Delta t$ to guarantee good estimates of the rocking response based on an extensive numerical campaign and a multivariable nonlinear regression is discussed. The coefficient of restitution $r$ is here assumed as an independent parameter [57]. The choice to keep $r$ independent allows the employment of any experimentally measured coefficient of restitution (e.g. [33, 65, 71]) or theoretically improved model (e.g. [47, 72–74]) to be adopted, which might differ from the one in Eq. (2) [33, 64, 65].

Furthermore, it has been found from preliminary analyses that the setting of the time step $\Delta t$ is influenced, beyond the contact stiffness ${k}_{n}$, as discussed in Section 2.2, and the coefficient of restitution $r$ [57], by the size and the aspect ratio of the block, i.e. by $R$ and $H/B$, respectively. Accordingly, the setting of the time step might be reasonably represented, similarly to what utilized in [57] for an akin problem, by the following simple function:

6 $$\Delta t={A}_{1}{\left(R \frac{H}{B}\right)}^{{A}_{2}}{k}_{n}^{{A}_{3}}\text{ln}\left(r\right)$$

where ${A}_{1}$, ${A}_{2}$, and ${A}_{3}$ are coefficient to be determined. It is here highlighted that such function will provide $\Delta t=0$ for $r=1$. This extreme case is out of interest for the present study as $r<1$ for all real cases. A suitable strategy for the identification of ${A}_{1}$, ${A}_{2}$, and ${A}_{3}$ is discussed in the following.

### Numerical campaign

An extensive numerical campaign is here carried out to estimate the coefficients ${A}_{1}$, ${A}_{2}$, and ${A}_{3}$ in Eq. (6). In total, 10 different block geometries with 3 different aspect ratios are considered, as specified in Table 1. Such geometries are chosen to have a reasonable replication of real rocking structures (namely cultural heritage structures). For each block geometry, 4 different values of ${k}_{n}$ are considered, i.e. 2.5e + 08, 5e + 08, 10e + 08, and 25e + 08 N/m^3^ (for later convenience labeled as k2.5, k5, k10, and k25, respectively), adopted in a reasonable range according to [57, 75]. Finally, for each case, 40 values of $\Delta t$ are considered within the range of 0.001 s, 0.002 s, …., 0.040 s.

**Table 1:** Blocks geometries employed in the numerical campaign

Block label	$2H$ [m]	$2B$ [m]	$R$ [m]	$H/B$ [–]
HB4_R0.62	1.2	0.3	0.62	4
HB4_R1.24	2.4	0.6	1.24	4
HB4_R2.48	4.8	1.2	2.48	4
HB4_R3.71	7.2	2.8	3.71	4
HB7_R1.06	2.1	0.3	1.06	7
HB7_R2.12	4.2	0.6	2.12	7
HB7_R4.24	8.4	1.2	4.24	7
HB10_R1.51	3.0	0.3	1.51	10
HB10_R3.02	6.0	0.6	3.02	10
HB10_R6.04	12.0	1.2	6.04	10

Accordingly, the numerical campaign here discussed is composed of 1600 numerical simulations of free rocking. Given the amplitude independence shown in Fig. 5, all these simulations have been conducted by adopting ${\theta }_{0}/\alpha =0.5$. According to [64, 76], a material density equal to 2600 kg/m^3^ has been here adopted for the blocks, while the density variability for common stone/masonry construction materials is consider negligible for the calibration purposes of this work. In all cases, the free rocking response is analysed for 30 s, or the minimum time needed to reach the maximum rocking angle of a cycle equal to $0.05{\theta }_{0}/\alpha$ if greater than 30 s.

**Fig. 6:**
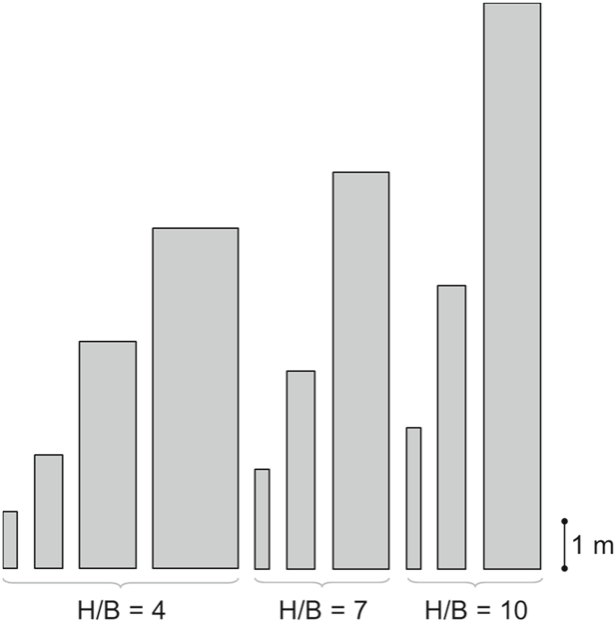
Blocks geometry proportions used in the numerical campaign. For actual sizes, refer to Table 1

To visualize the various blocks geometries considered, their proportions are highlighted in Fig. 6, organized with the same layout of Table 1.

### Regression for the setting of the time step

**Fig. 7:**
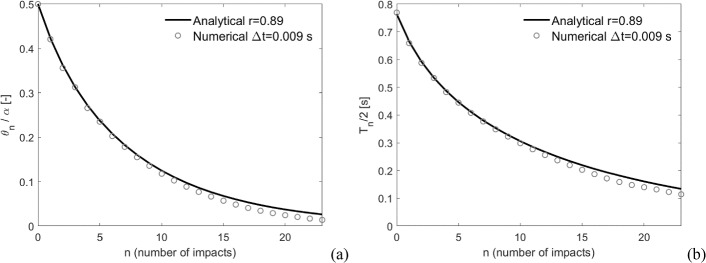
Example of comparison between analytical and numerical solutions, in terms of **a** normalized rocking angle and **b** half rocking period, along with the number of impacts, for r = 0.89 and $\Delta t$=0.009 s (case HB4_R0.62_k2.5)

The comparison against the analytical solution in Eq. (3) is performed in terms of rocking angle and half rocking period, for a significant number of impacts $N$, which is here identified as the number of impacts needed to reach a rocking angle lower than $0.05{\theta }_{0}/\alpha$. An example of comparison between analytical and numerical solutions is shown in Fig. 7, in terms of normalized rocking angle (Fig. 7a) and half rocking period (Fig. 7b), along with the number of impacts for a certain $r$ and a certain $\Delta t$.

An error measurement between analytical and numerical solutions is here introduced. Firstly, the root-mean-square error of the rocking angle normalized on the initial angle (${e}_{\theta /\alpha }$), and of the half rocking periods normalized on the initial period (${e}_{T/2}$) is computed, for a significant number of impacts $N$, as:

7 $$\begin{aligned} & {e}_{\theta }=\frac{1}{{\widehat{\theta }}_{0}}\sqrt{\frac{1}{N}\sum_{\text{n}=0}^{N}{\left|{\widehat{\theta }}_{\text{n}}-{\widetilde{\theta }}_{\text{n}}\right|}^{2}} , \\ & {e}_{T}=\frac{1}{{\widehat{T}}_{0}/2}\sqrt{\frac{1}{N}\sum_{\text{n}=0}^{N}{\left|{\widehat{T}}_{\text{n}}/2-{\widetilde{T}}_{\text{n}}/2\right|}^{2}} \end{aligned}$$

where the symbol $\hat{\blacksquare}$ denotes values obtained through the analytical solution, and the symbol $\tilde{\blacksquare}$ denotes values obtained through the numerical solution. Secondly, these two error measures are combined into a unique global measurement of the relative error ${e}_{G}$, defined as:

8 $${e}_{G}=\sqrt{{e}_{\theta }^{2}+{e}_{T}^{2}}$$

This global measurement of the relative error ${e}_{G}$ is here used within a sort of optimization problem, where the optimal $\Delta t$ is selected as the case with the lowest ${e}_{G}$, aimed at investigating the optimal $\Delta t$ to be used in numerical simulations given the block geometry, $r$, and ${k}_{n}$. Accordingly, ${e}_{G}$ is computed for each of the 40 considered $\Delta t$s, for each block listed in Table 1, for each value of contact stiffness ${k}_{n}$, and for coefficients of restitution $r$ varying within the range 0.80, 0.81, …, 0.99.

**Fig. 8:**
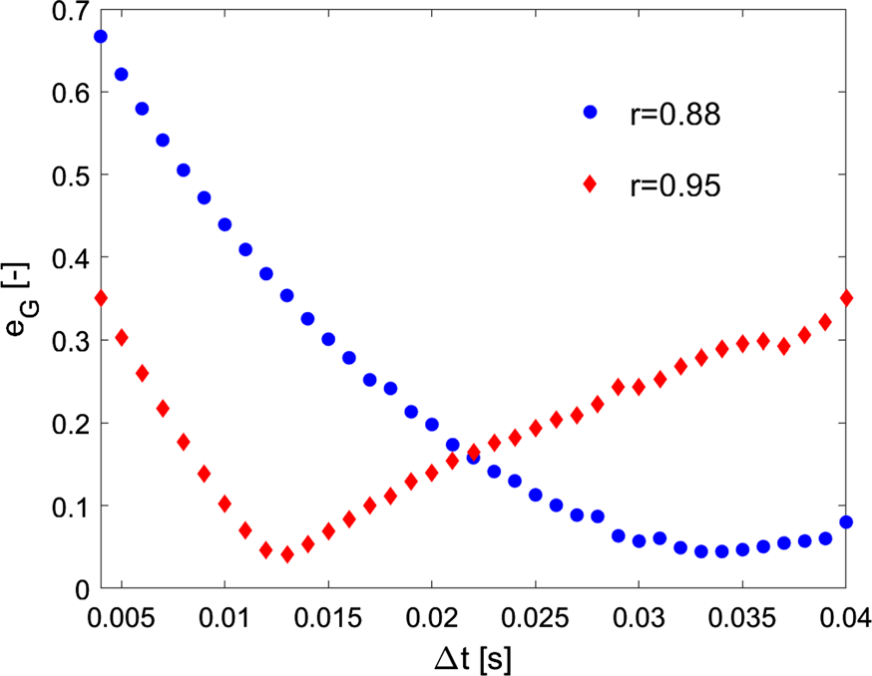
Distribution of ${e}_{G}$ along with the considered time steps: examples of a lower value ($r=0.88$) and a higher value ($r=0.95$) of coefficient of restitution (case HB7_R2.12_k2.5)

Examples of distribution of ${e}_{G}$ along with the considered time steps are shown in Fig. 8, where the error trends between two different values of coefficient of restitution (a lower value 0.88, and a higher value 0.95) are compared. As it can be noted, the error ${e}_{G}$ reaches small values for a range of $\Delta t$s. In particular, it is observed that lower values of $r$ (e.g., 0.88) show smoother trends of ${e}_{G}$, i.e., a wider range of $\Delta t$ is characterized by small errors, while higher values of $r$ (e.g., 0.95) show steeper trends of ${e}_{G}$, i.e., a narrower range of $\Delta t$ is characterized by small errors. By way of example, with reference to Fig. 8, an error ${e}_{G}\le 10\%$ is obtained with $0.10 \, s\le \Delta t\le 0.17 \, s$ for $r=0.95$, while with $0.26 \, s\le \Delta t\le 0.40 \, s$ for $r=0.95$ (considering also that values of $\Delta t>0.40\,s$ have not been here considered). Accordingly, the adoption of a $\Delta t$ in a neighborhood of the optimal $\Delta t$ would still guarantee accurate results. This is particularly true for lower values of coefficient of restitution, i.e. for values of $r$ expected in real historical structures with, e.g., mortar joints and/or defects.

Moreover, it should be pointed out that the present approach is based on the HHT method and the convergence within each increment is not guaranteed. Indeed, for highly nonlinear problems (e.g. damage constitutive laws, contact cohesion, etc.) convergence may not be found within a time increment. This might be overcome by reducing the time increment (only for the non-converged increment, e.g., by 50%) to obtain a solution. This aspect may affect locally (in time) the dissipative properties of the solution, and the analysis report should be checked to judge the quality of the response. In any case, in all the simulations considered in this paper, non-converged increments have not been recorded.

In the following, the optimal $\Delta t$ has been chosen as the one with the minimum value of ${e}_{G}$ (anyway, considered only when ${e}_{G}\le 7\%$ to exclude the extreme cases). In the following, the so-computed optimal $\Delta t$s are referred to as “measured optimal $\Delta t$”.

**Fig. 9:**
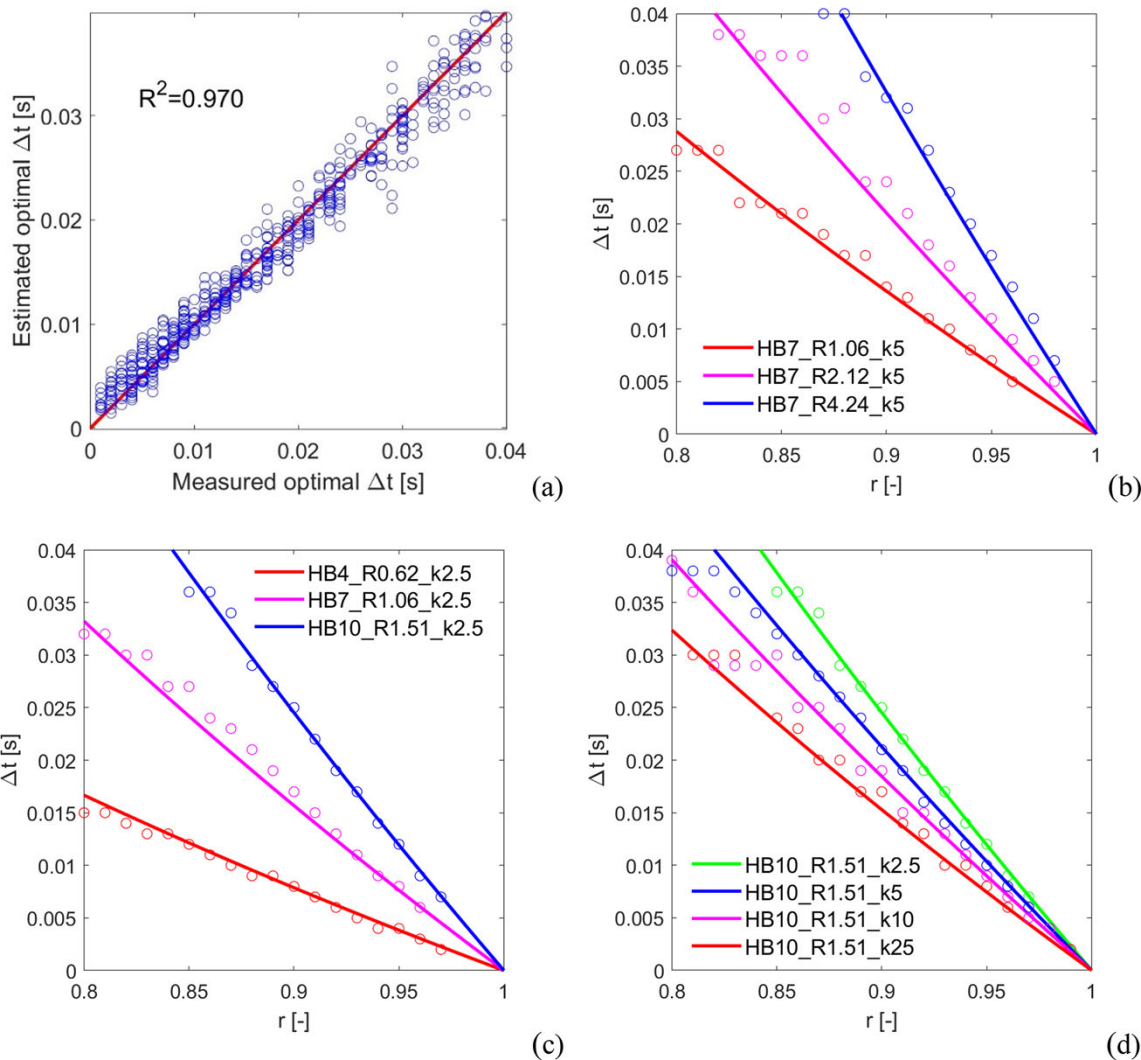
Results of the multivariable nonlinear regression analysis. **a** Estimated versus measured optimal time step plot (coefficient of determination ${\text{R}}^{2}=0.970$). Comparison of estimated (solid lines) versus measured (hollow circles) time steps along with $r$ by varying **b** the block size, i.e. $R$, **c** the block aspect ratio, i.e. $H/B$, as well as slightly the block size $R$, and **d** the contact stiffness, i.e. ${k}_{n}$

A multivariable nonlinear regression analysis is then performed using the results of the numerical campaign and, in particular, the measured optimal $\Delta t$. As a result, the coefficients of Eq. (6) have been determined, and the resulting analytic formula for the setting of the time step (in the following, referred to as “estimated optimal $\Delta t$”), with a coefficient of determination ${\text{R}}^{2}=0.970$, is:

9 $$\Delta t=-2.238{\left(R \frac{H}{B}\right)}^{0.629}{k}_{n}^{-0.205}\text{ln}\left(r\right)$$

with $\Delta t$ in s, $R$ in m, and ${k}_{n}$ in N/m^3^ (being $H/B$ and $r$ dimensionless). It should be pointed out that $R$ and $H/B$ are raised to the same power as, after an initial investigation, it has been found that even if two different power parameters were supposed they would practically assume the same value during the multivariable nonlinear regression. Additionally, for rectangular cuboid blocks, Eq. (9) can be also written in terms of frequency parameter $p$ and slenderness $\alpha$ as:

10 $$\Delta t=-7.851{\left( \frac{\text{cot}\alpha }{{p}^{2}}\right)}^{0.629}{k}_{n}^{-0.205}\text{ln}\left(r\right)$$

with $\Delta t$ in s, $p$ in Hz, $\alpha$ in rad, and ${k}_{n}$ in N/m^3^ (being $r$ dimensionless). The results of the multivariable nonlinear regression analysis are shown in Fig. 9. In particular, the estimated versus measured optimal $\Delta t$ plot is shown in Fig. 9a, where an overall good agreement between estimated and measured optimal $\Delta t$ can be observed (as also confirmed by the rather high coefficient of determination). This agreement is further highlighted by the comparison between estimated and measured time steps along with $r$ by varying the block size, i.e. $R$ (Fig. 9b), the block aspect ratio, i.e. $H/B$ (Fig. 9c), and the contact stiffness, i.e. ${k}_{n}$ (Fig. 9d).

Interestingly, it is found that by increasing the block size $R$ for a fixed $r$, also the optimal $\Delta t$ increases (as it could be deduced by the coefficients in Eq. (9) and in Fig. 9b). This aspect is particularly appealing for real case applications, such as rocking of monuments and cultural heritage structures, as it allows faster dynamic simulations for larger structures.

### Post validation

The validation of the regression function in Eq. (9) is conducted a posteriori with cases not included in the training set by adopting the blocks geometry in Table 2 with 3 different values of ${k}_{n}$, i.e. 4e + 08, 7e + 08, and 12e + 08 N/m^3^ (for convenience, labeled as k4, k7, and k12, respectively). It is here highlighted that such cases represent intermediate cases within the range of parameters investigated.

**Table 2:** Blocks geometry for post validation

Block label	$2H$ [m]	$2B$ [m]	$R$ [m]	$H/B$ [–]
HB6_R0.91	1.8	0.3	0.91	6
HB8.5_R2.57	5.1	0.6	2.57	8.5

**Fig. 10:**
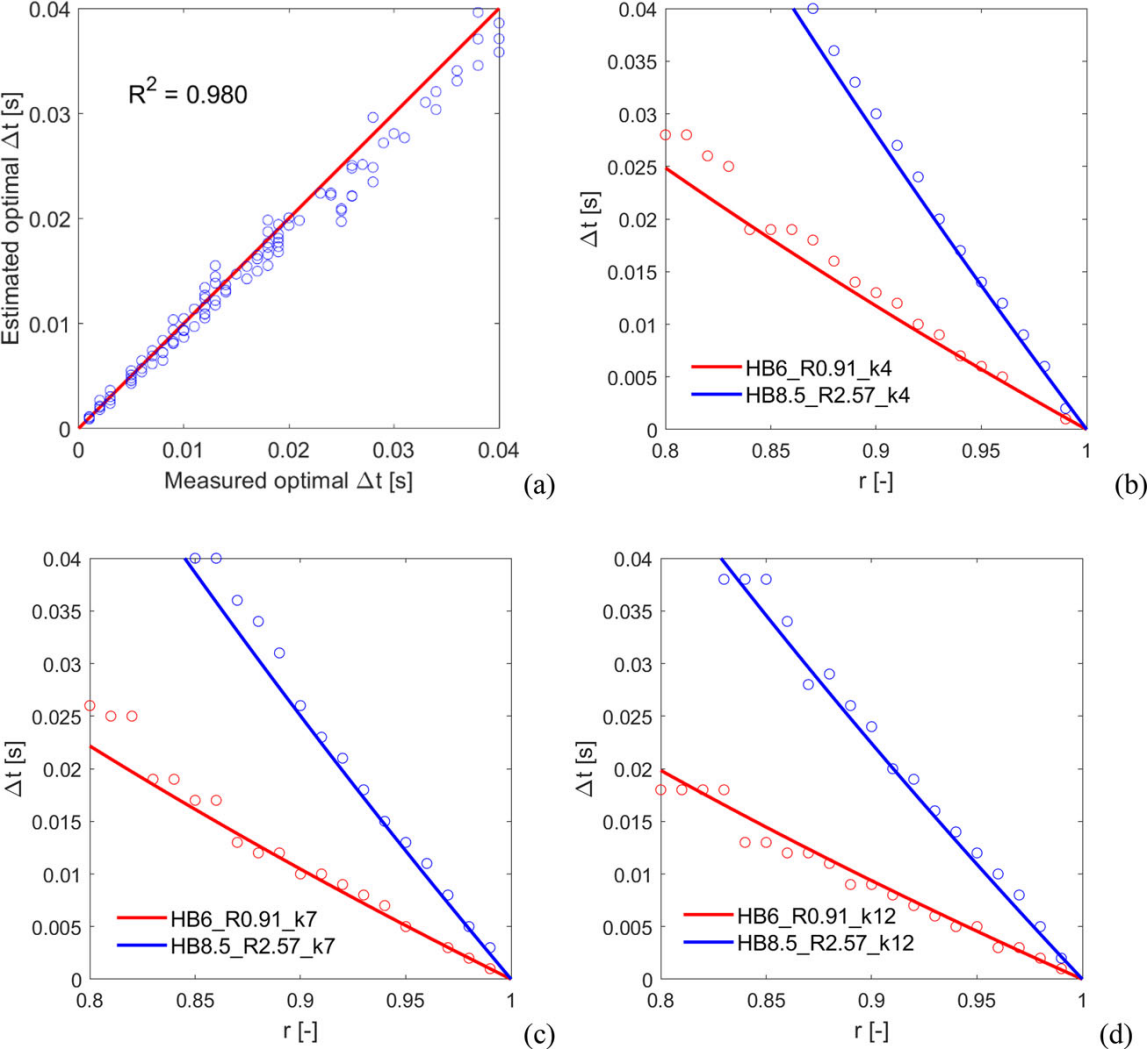
Results of the a posteriori validation. **a** Estimated versus measured optimal time step plot (coefficient of determination ${\text{R}}^{2}=0.980$). Comparison of estimated (solid lines) versus measured (hollow circles) time steps along with $r$ for **b** ${k}_{n}=$4e + 08 N/m^3^, **c** ${k}_{n}=$7e + 08 N/m^3^, and **d** ${k}_{n}=$12e + 08 N/m^3^

The comparison of the predictions of Eq. (9) with the numerical results obtained with the blocks in Table 2 is shown in Fig. 10. As it can be seen in Fig. 10a, Eq. (9) well predicts the optimal $\Delta t$ for cases not in the training set, as also confirmed by the coefficient of determination ${\text{R}}^{2}=0.980$. This good prediction is also highlighted by the comparison between estimated and measured time steps along with $r$ for k4 (Fig. 10b), k7 (Fig. 10c), and k12 (Fig. 10d). Accordingly, the analytic formula for the setting of the time step in Eq. (9) appears to be robust, accurate, and reliable. In the following, it is thus used to reproduce experiments.

## Comparison with experimental tests

In this section, the outcomes of the experimental campaigns in [15, 64] are used to compare the results of the present computational approach. Particularly, free rocking and harmonic loading cases are treated in a deterministic sense [64], while earthquake-like loading cases are treated in a statistical sense (according to [15]). For each case, the time step $\Delta t$ for the present computational approach is adopted according to Eq. (9). In the following, the $\Delta t$ adopted is symbolized in the graphs as “t” followed by the digits after the decimal (e.g. $\Delta t=0.0123$ s is concisely depicted as “t0123”).

### Experimental campaign by Peña et al. (2008)

In this section, the outcomes of the experimental campaign discussed in [64] are used as reference. In particular, three specimens are here considered, see Table 3. It is worth highlighting that the dimensions of these specimens are considerably smaller than the range of block dimensions adopted in the numerical campaign aimed at the setting of the time step (Table 1). Three values of contact stiffness, i.e. k2.5, k5, and k10, are considered in the numerical solutions, while the coefficients of restitution provided in [64] are utilized for the analytical solutions and as input for Eq. (9).

**Table 3:** Details of the specimens considered, from [64]

Specimen	$2H$ [m]	$2B$ [m]	$H/{\text {B}}$	Test cases
1	1.0	0.25	4.0	Free rocking + harmonic sinusoidal excitation with frequency 3.3 Hz, amplitude 6 mm, and duration 20 s
2	1.0	0.17	5.9	Free rocking + harmonic sinusoidal excitation with frequency 5.0 Hz, amplitude 5 mm, and duration 10 s
3	1.0	0.12	8.3	Free rocking + artificial ground motion n. 18, load factor: 0.5, see [64] for more details

**Fig. 11:**
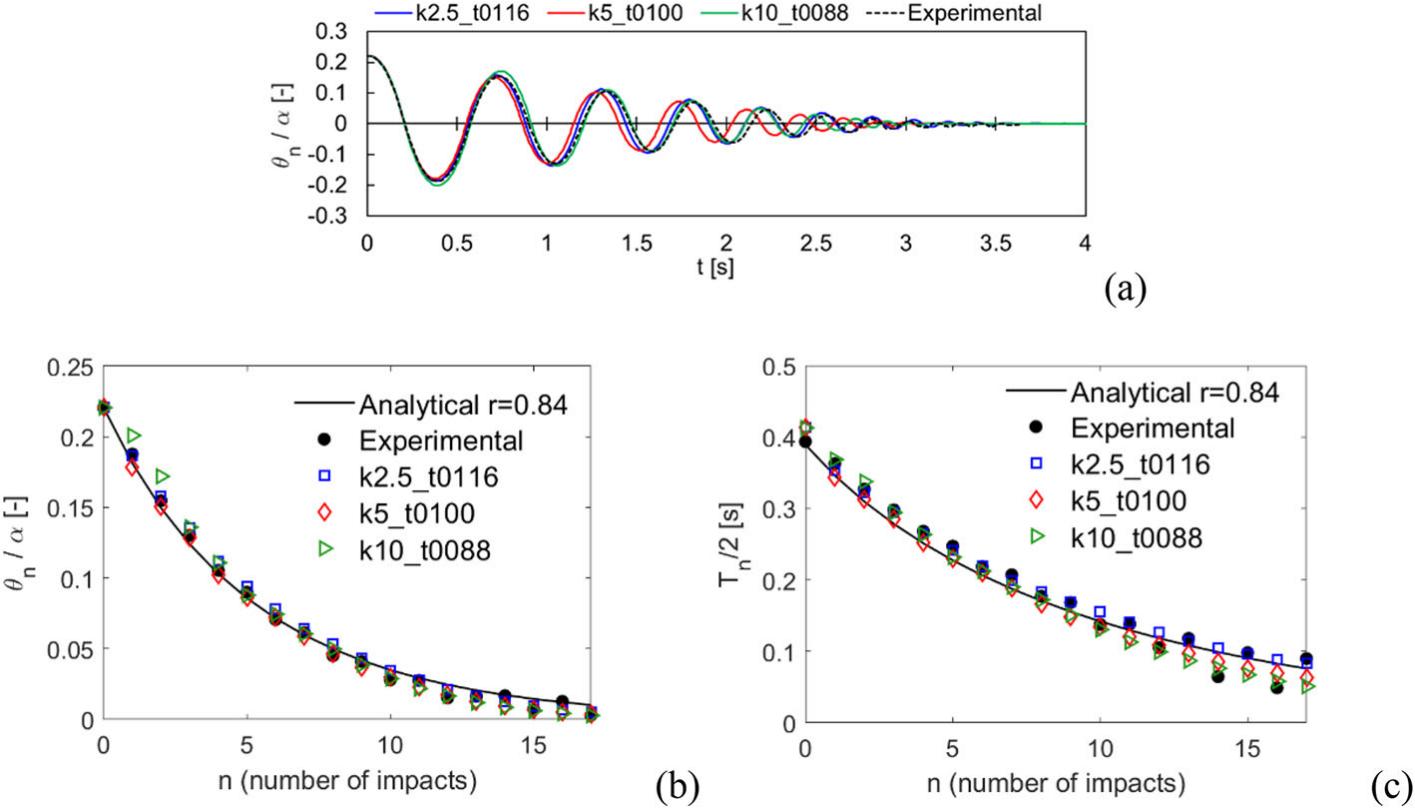
Comparison with the experimental tests by Peña et al. [64] for Specimen 1, free rocking. **a** Normalized rocking angle time history. **b** Normalized rocking angle and **c** half rocking period along with the number of impacts

The results comparison for Specimen 1 in free rocking is shown in Fig. 11, in terms of normalized rocking angle time history (Fig. 11a), normalized rocking angle (Fig. 11b) and half rocking period (Fig. 11c) along with the number of impacts. An overall good agreement between numerical and experimental results is observed, both following the analytical solution. All the three considered values of contact stiffness show consistent results, although the case k5 shows a small difference especially in the period (Fig. 11a). Anyway, it should be pointed out that the case k5 is still in good agreement with the analytical rocking period (Fig. 11c).

**Fig. 12:**
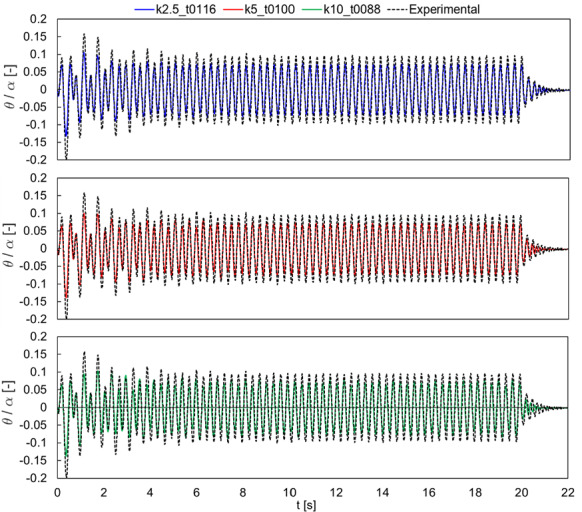
Comparison with the experimental tests by Peña et al. (2008) [64] for Specimen 1, harmonic loading. Normalized rocking angle time histories

The comparison of Specimen 1 with harmonic excitation is shown in Fig. 12, in terms of normalized rocking angle time histories. The three considered values of contact stiffness show very similar results, with peak amplitudes always slightly smaller than the experimental result. It is here worth to mention that a similar trend was also observed in [57]. Nevertheless, the free rocking behavior (i.e., after 20 s) is accurately predicted by all cases.

**Fig. 13:**
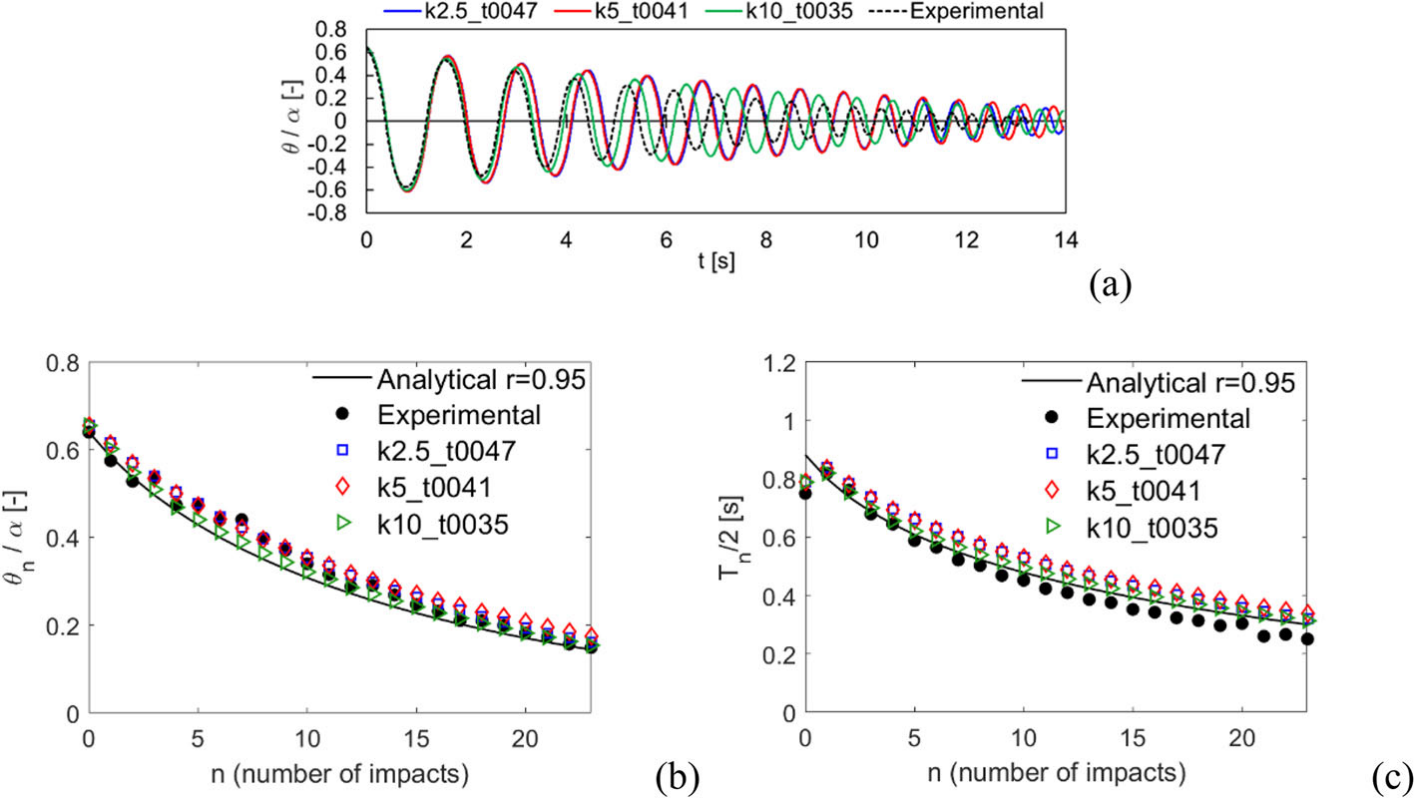
Comparison with the experimental tests by Peña et al. (2008) [64] for Specimen 2, free rocking. **a** Normalized rocking angle time history. **b** Normalized rocking angle and **c** half rocking period along with the number of impacts

**Fig. 14:**
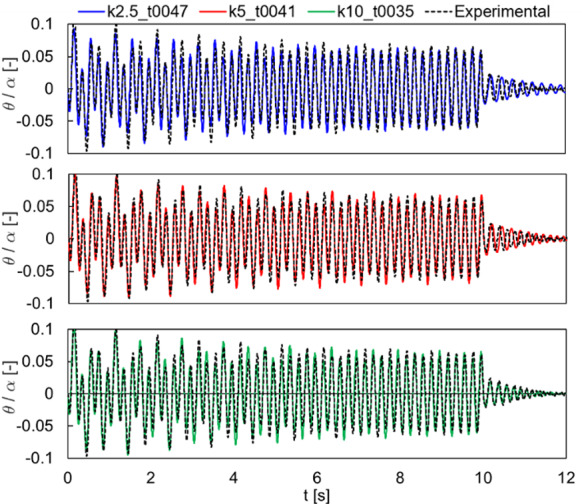
Comparison with the experimental tests by Peña et al. (2008) [64] for Specimen 2, harmonic loading. Normalized rocking angle time histories

The comparison of the results for Specimen 2 are shown in Figs. 13 and 14 for free rocking and harmonic excitation, respectively. Free rocking time histories are more dispersed than the previous specimen (Fig. 13a), although the rocking angle and period decay along with the number of impacts is consistent with the analytical solution (Fig. 13b–c). Indeed, small differences between experimental results and the analytical solution can be noted for both rocking angle (Fig. 13b) and rocking period (Fig. 13c). It is worth noting that numerical results are anyway included in between this range of variability. Note that numerical time histories have been shifted towards left to agree with the initial rocking period measured in the experiment, which is shorter than the analytical prediction. The harmonic excitation response comparison (Fig. 14) highlights an overall good agreement for all the three considered cases, also for the free rocking behavior (i.e., after 10 s).

**Fig. 15:**
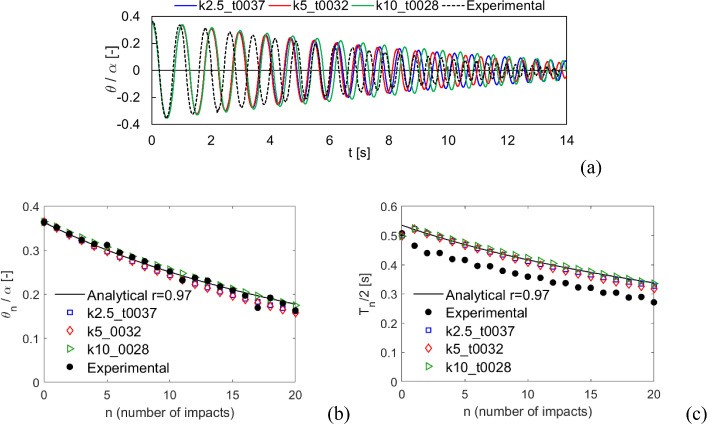
Comparison with the experimental tests by Peña et al. (2008) [64] for Specimen 3, free rocking. **a** Normalized rocking angle time history. **b** Normalized rocking angle and **c** half rocking period along with the number of impacts

The results comparison for Specimen 3 free rocking is shown in Fig. 15. The numerical time histories of the 3 cases are consistent between each other (Fig. 15a) and show a rocking angle decay close to the experimental one. However, the rocking periods (except for the initial one) appear to be significantly different between experimental and numerical. A similar shift in the rocking periods was also observed in [57]. By looking at the rocking angle (Fig. 15b) and period (Fig. 15c) along with the number of impacts, is it possible to note that, on the one hand, an overall good agreement of the rocking angle (Fig. 15b) is observed between experimental, analytical, and numerical results, while, on the other hand, numerical results fit pretty well the analytical solution for half rocking periods, being the experimental periods systematically lower than the other solutions. This again shows the good consistency of the present computational approach with the reference analytical solution.

**Fig. 16:**
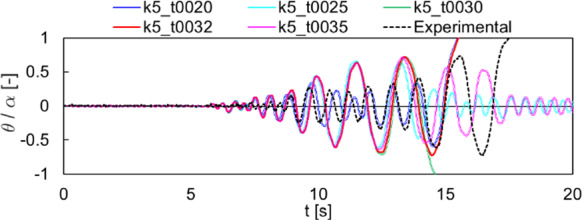
Comparison with the experimental tests by Peña et al. (2008) [64] for Specimen 3, earthquake-like loading. Normalized rocking angle time histories

The results comparison for Specimen 3 subjected to an artificial ground motion are shown in Fig. 16. For this test, only the case k5 has been considered, which is characterized by an optimal time step $\Delta t=0.0032$ s (t0032). In such experimental test, the specimen collapses. This outcome is also obtained with the reference numerical solution (t0032), although collapse is reached few seconds before with respect to the experiment and the numerical normalized rocking angle time history differs significantly from the experiment. To check the sensitivity of the time step in the collapse response of this specimen, time steps equal to 0.0020, 0.0025, 0.0030, 0.0035 s are also shown in Fig. 16 for the sake comparison (with k5 in each scenario). As it can be noticed, collapse is obtained with t0020, t0030, and t0032, while no collapse is observed for t0025 and t0035. Additionally, a large variability of numerical rocking angle time histories is observed, although the adopted time steps are pretty similar. Accordingly, no clear trend can be deduced from Fig. 16, as the response appears chaotic. In this regard, the rocking response to earthquake-like ground motions is discussed in a statistical sense in the next subsection, according to [15].

### Experimental campaign by Bachmann et al. (2018)

**Fig. 17:**
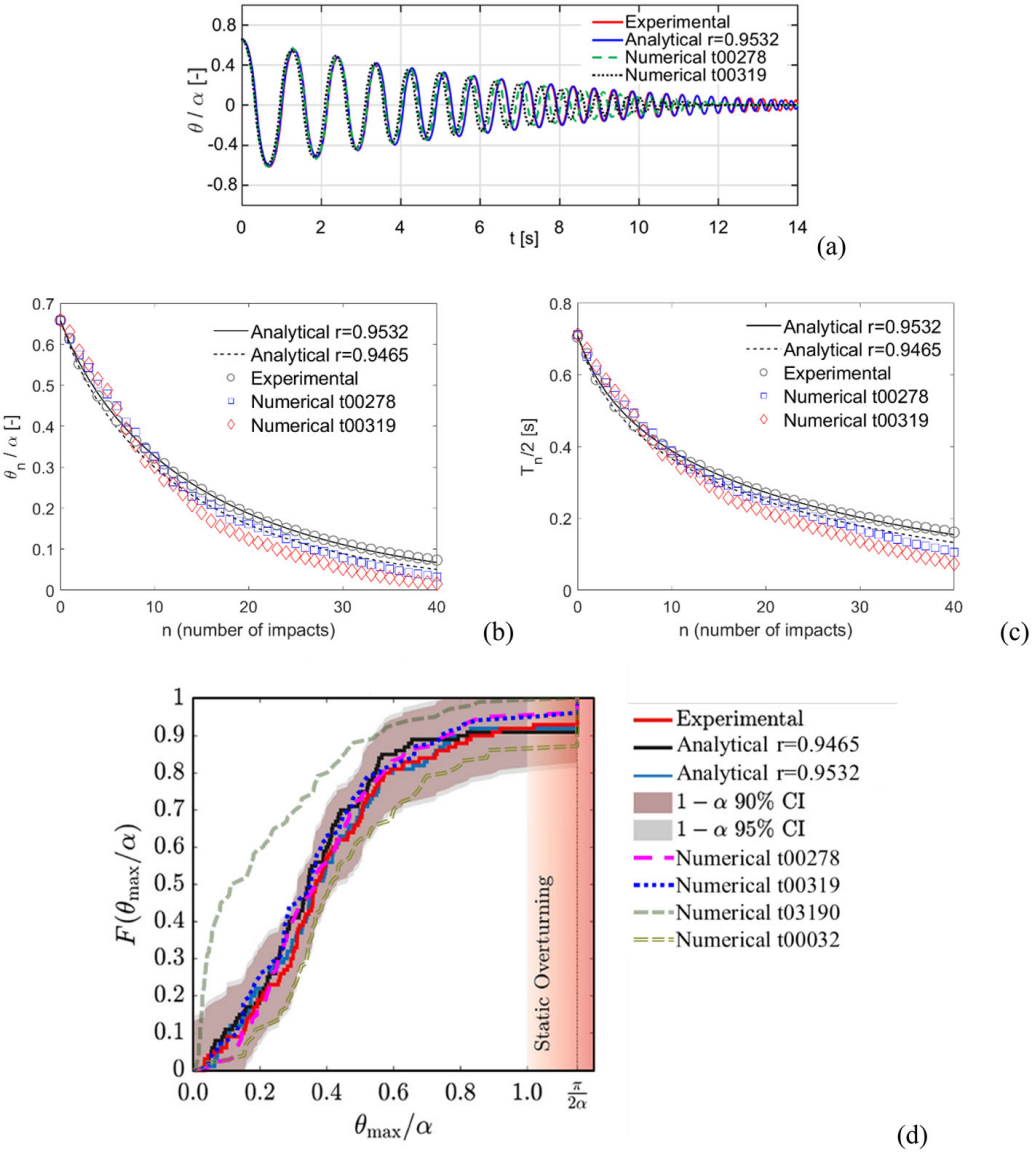
Comparison with the experimental tests by Bachmann et al. [15]. **a** Free rocking normalized rocking angle time history, adapted from [15]. **b** Normalized rocking angle and **c** half rocking period along with the number of impacts. **d **Earthquake-like input, Case Lefkada 2H = 10 m, cumulative distribution functions of the maximum normalized rocking angle for 100 tests (adapted from [15])

In this section, the experimental campaign discussed in [15] is considered and compared (Fig. 17) with the computational approach here proposed. Firstly, an equivalent cuboid solid block ($2H = 0.609 \, \text{m}, \, 2B = 0.09135 \, \text{m}$) is deduced from the value of frequency parameter $p$ identified experimentally (4.8883 Hz) and $\text{tan}\alpha =0.15$ [15]. In particular, an equivalent $R$ is obtained from $p$, considering a rectangular cuboid block, and $B$ and $H$ are then determined according to $\alpha$. By considering the coefficient of restitution determined experimentally (i.e., 0.9532), and the one coming from the classical rocking theory [16] (i.e., 0.9465), two optimal time steps, i.e. t00278 and t00319, respectively, are set according to Eq. (9), by considering a contact stiffness k5.

The comparison between the experimental, analytical, and numerical results for a free rocking test is shown in Fig. 17a in terms of normalized rocking angle time history, and in Fig. 17b–c in terms of normalized rocking angle and half rocking period, respectively, along with the number of impacts. Although the need of resorting to an equivalent cuboid solid block, numerical results appear in a good agreement with the experimental/analytical ones, for both time steps considered, with less accuracy in the last part of the free rocking response (characterized by small rocking angles). Considering that also in this case the equivalent block dimensions are significantly smaller than the range of dimensions adopted in the numerical campaign for the setting of the time step (Table 1), such results are promising.

**Fig. 18:**
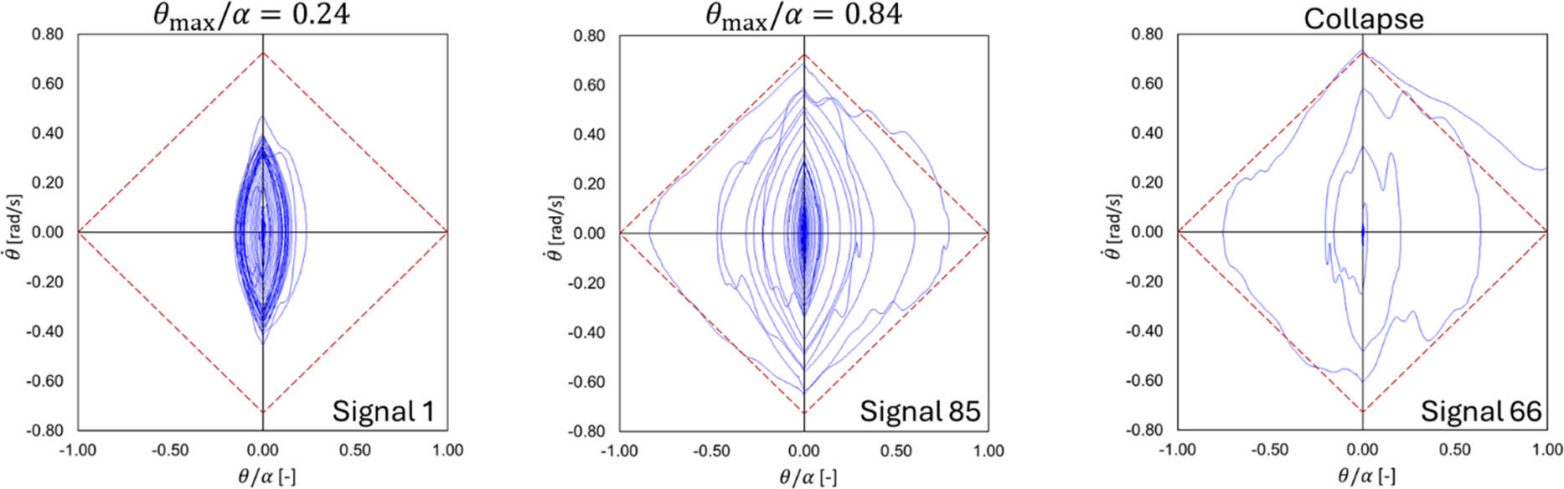
Phase portraits for different simulations with t00278

The “Case Lefkada 2H = 10 m” described in [15] has been considered here (as it presents a full range of normalized rocking angles, including also collapses). This case conceives the application of 100 different artificial ground motions, generated by a stochastic model to match the physical characteristics of the 2003 Lefkada earthquake, subsequently scaled in time to indirectly increase the dimensions of the specimen. The actual accelerograms recorded on the shaking table [15] have been used as input in the numerical simulations. The results of these simulations are shown and compared with experimental and analytical results in Fig. 17d in terms of cumulative distribution functions of the maximum normalized rocking angle ($F({\theta }_{max}/\alpha )$) for the 100 tests. In Fig. 17d, adapted from [15], the 90% and 95% nonparametric confidence intervals (CI) are also reported for the experimental cumulative distribution function (the interested reader is referred to [15] for more details). As it can be noted, the cumulative distribution functions obtained numerically with t00278 and t00319 fit very well the experimental/analytical ones, being also included within the aforementioned CIs. Three phase portraits for conditions far from collapse (Signal 1, with ${\theta }_{max}/\alpha =0.24$), near to collapse (Signal 85, with ${\theta }_{max}/\alpha =0.84$), and collapse (Signal 66) are shown in Fig. 18, together with the rhomboidal separatrix [70] (red dotted lines), delimitating stable paths of rocking motion (being the maximum angular velocity evaluated as $2p\text{sin}\alpha /2$). In particular, by comparing the phase portraits of Signal 85 and Signal 66, it is further highlighted the randomness between collapse and no collapse conditions, which strongly depends on the signal and the phase between current rocking angle and the signal.

As a counterexample, the numerical results with time steps very far from the optimal one (i.e., t03190, which is 10 times greater than the highest mentioned before, and t00032, which is 10 times smaller) show cumulative distribution functions (Fig. 17d) considerably far from the others. Indeed, the case t03190 appears completely outside from the considered CIs, while the case t00032 results on the CIs frontier for most of the curve and in the other side of the envelope with respect to the t03190 case. This outcome highlights the efficacy of the present approach in predicting the rocking response subjected to ground motions in a statistical sense, and the proposed setting of the time step appears robust and general.

## Conclusions

In this paper, the possibility of utilizing an implicit time integration scheme with numerical dissipation and without any damping model to simulate rocking blocks has been investigated. According to the present computational approach, a rocking block has been idealized as a solid body interacting with its foundation through a contact-based formulation. The well-known HHT method, set to optimally treat dissipation in contact problems, has been employed, being the numerical dissipation governed by the time step. The rocking dissipative phenomenon at impacts appeared to be accurately predicted by the proposed computational approach without the use of any damping model.

A broad numerical campaign has been conducted to define a regression law in analytic form for the setting of the optimal time step. Such law has been found to be dependent on the block size and aspect ratio, the contact stiffness, as well as the coefficient of restitution selected. The so-obtained regression law appeared accurate and an a posteriori validation with cases not in the training dataset confirmed the effectiveness and the robustness of the approach. Interestingly, it has been found that by increasing the block size also the optimal time step increases (so allowing fast dynamic simulations even for large-scale structures). In particular, it has been found that rocking blocks with sizes of interest for structural engineering (namely cultural heritage structures) can be simulated with time steps within 10^–3^ ÷ 10^–1^ s, so allowing very fast computations.

Finally, the comparison with available experimental tests highlighted the efficacy of the present computational approach for free rocking and harmonic loading cases (in a deterministic sense), and for earthquake-like loading cases (in a statistical sense, i.e., in terms of cumulative distribution functions).

Future developments will concern the extension of the present computational approach to multi-block rocking structures, e.g., by exploiting the concept of dynamically equivalent rocking structures [23] to set the time step in a straightforward way.

## Data Availability

The datasets analyzed during the current study are available in an open data repository.
